# Identification of a cytokine profile in serum and cerebrospinal fluid of pediatric and adult spinal muscular atrophy patients and its modulation upon nusinersen treatment

**DOI:** 10.3389/fncel.2022.982760

**Published:** 2022-08-11

**Authors:** Silvia Bonanno, Paola Cavalcante, Erika Salvi, Eleonora Giagnorio, Claudia Malacarne, Marco Cattaneo, Francesca Andreetta, Anna Venerando, Viviana Pensato, Cinzia Gellera, Riccardo Zanin, Maria Teresa Arnoldi, Claudia Dosi, Renato Mantegazza, Riccardo Masson, Lorenzo Maggi, Stefania Marcuzzo

**Affiliations:** ^1^Neurology IV - Neuroimmunology and Neuromuscular Diseases Unit, Fondazione IRCCS Istituto Neurologico Carlo Besta, Milan, Italy; ^2^Neuroalgology Unit, Fondazione IRCCS Istituto Neurologico Carlo Besta, Milan, Italy; ^3^Ph.D. Program in Neuroscience, University of Milano-Bicocca, Monza, Italy; ^4^Unit of Medical Genetics and Neurogenetics, Fondazione IRCCS Istituto Neurologico Carlo Besta, Milan, Italy; ^5^Developmental Neurology Unit, Fondazione IRCCS Istituto Neurologico Carlo Besta, Milan, Italy

**Keywords:** SMA, nusinersen, immune system, multisystemic, biomarker

## Abstract

**Background and objectives:**

Multisystem involvement in spinal muscular atrophy (SMA) is gaining prominence since different therapeutic options are emerging, making the way for new SMA phenotypes and consequent challenges in clinical care. Defective immune organs have been found in preclinical models of SMA, suggesting an involvement of the immune system in the disease. However, the immune state in SMA patients has not been investigated so far. Here, we aimed to evaluate the innate and adaptive immunity pattern in SMA type 1 to type 3 patients, before and after nusinersen treatment.

**Methods:**

Twenty one pediatric SMA type 1, 2, and 3 patients and 12 adult SMA type 2 and 3 patients were included in this single-center retrospective study. A Bio-Plex Pro-Human Cytokine 13-plex Immunoassay was used to measure cytokines in serum and cerebrospinal fluid (CSF) of the study cohort before and after 6 months of therapy with nusinersen.

**Results:**

We detected a significant increase in IL-1β, IL-4, IL-6, IL-10, IFN-γ, IL-17A, IL-22, IL-23, IL-31, and IL-33, in serum of pediatric and adult SMA patients at baseline, compared to pediatric reference ranges and to adult healthy controls. Pediatric patients showed also a significant increase in TNF-α and IL-17F levels at baseline. IL-4, IFN-γ, Il-22, IL-23, and IL-33 decreased in serum of pediatric SMA patients after 6 months of therapy when compared to baseline. A significant decrease in IL-4, IL-6, INF-γ, and IL-17A was detected in serum of adult SMA patients after treatment. CSF of both pediatric and adult SMA patients displayed detectable levels of all cytokines with no significant differences after 6 months of treatment with nusinersen. Notably, a higher baseline expression of IL-23 in serum correlated with a worse motor function outcome after treatment in pediatric patients. Moreover, after 6 months of treatment, patients presenting a higher IL-10 concentration in serum showed a better Hammersmith Functional Motor Scale Expanded (HFMSE) score.

**Discussion:**

Pediatric and adult SMA patients show an inflammatory signature in serum that is reduced upon *SMN2* modulating treatment, and the presence of inflammatory mediators in CSF. Our findings enhance SMA knowledge with potential clinical and therapeutic implications.

## Introduction

Spinal muscular atrophy (SMA) is an autosomal recessive disorder caused by mutations in survival motor neuron (*SMN1*) gene, resulting in a truncated SMN protein responsible for degeneration of brain stem and spinal motor neurons (Hamilton and Gillingwater, 2013). Even with the same disease-causing mutation, SMA phenotype varies widely from very severe infantile forms to mild adult-onset (Mercuri et al., 2012; Finkel et al., 2015). The *SMN2* copy number ranges from 0 to ≥4 copies across the SMA population, where a higher *SMN2* copy number is usually associated with a milder phenotype, establishing *SMN2* as the best renowned prognostic biomarker so far (Wirth, 2021). For this reason, increasing SMN protein production by *SMN2* is the target of two out of the three recently approved therapies for the treatment of SMA, being the third directed to reintroduce *SMN1* gene (Finkel et al., 2017; Mendell et al., 2017; Darras et al., 2021; Mercuri et al., 2021). Antisense oligonucleotide nusinersen (Spinraza^®^) extends life expectancy, promotes the gaining of unexpected motor milestones for pediatric patients (Chiriboga, 2017), and improves motor function in adult SMA (Maggi et al., 2020). However, like the other therapies, it does not fully recover the phenotypes yet, and other targets of potential complementary therapeutic strategies are under investigation (Chaytow et al., 2021).

*SMN1* has ubiquitous expression in the organism, where it critically regulates several developmental and housekeeping cellular pathways (Chaytow et al., 2018). Therefore, it is not surprising that, despite the predominant susceptibility of motor neurons (Tosolini and Sleigh, 2017), evidence of a multisystem involvement is emerging in SMA. Systemic pathology is more evident in severe SMA-type 1 patients, but it is gaining importance with the advent of new disease-phenotypes due to SMN-rescue therapies, which are modifying SMA natural history (Yeo and Darras, 2020).

In this context, few reports about a potential dysregulation of the immune system in SMA have been previously reported in patients (Ryniewicz and Pawińska, 1978; Migaj et al., 1986) and deepened in preclinical animal model of the disease (Deguise and Kothary, 2017; Wan et al., 2018).

Recently, several studies highlighted the prominent role of immune activation in neurodegenerative diseases, including motor neuron disorders such as amyotrophic lateral sclerosis, where anti-inflammatory strategies are under investigation as suitable therapies (Crisafulli et al., 2018). However, the contribution of the immune system and inflammation to SMA pathogenesis, and its potential significance as a therapeutic target, is still unknown. Nonetheless, in the context of emerging therapies for SMA, the need for tools complementary to the clinical evaluation to improve patients’ management and clinicians’ decision making, is of relevance. For instance, the increasing use of *SMN1* gene replacement treatment through adeno-associated virus 9 vectors demands a better understanding of the immunological mechanisms underlying the disease, for proper safety assessment.

Here, we investigated for the first time the immune system involvement in SMA pathogenesis through a comprehensive profiling of cytokines in serum and cerebrospinal fluid (CSF) of SMA type 1 to type 3 patients, covering the wide disease spectrum from childhood to adulthood, before and after nusinersen treatment. The results of our analysis indicated an immune activation at peripheral level, modulated in an anti-inflammatory manner after 6 months of nusinersen treatment, and the presence of neuroinflammation, in pediatric and adult SMA patients. By identifying innate and adaptive immune mediators implicated in SMA, and modulated by nusinersen, our work contributes to the understanding of SMA pathogenesis and, prospectively, to the clinical management of pediatric and adult SMA patients.

## Materials and methods

### Patients and healthy controls

This is a single-center retrospective study including a cohort of 21 clinically and genetically defined pediatric SMA type 1, 2, and 3 patients followed-up at the Developmental Neurology Unit, 12 adult SMA type 2 and 3 patients followed-up at the Neurology IV - Neuroimmunology and Neuromuscular Diseases Unit of Fondazione IRCCS Istituto Neurologico Carlo Besta (Milan, Italy), and 11 adult healthy controls for the comparison of serum cytokine levels. Patients were genetically assessed at the Unit of Medical Genetics and Neurogenetics of Fondazione IRCCS Istituto Neurologico Carlo Besta. The study cohort consisted of SMA patients treated with nusinersen starting from December 2018, for at least 6 months, which corresponds to the loading period of treatment. Exclusion criteria were: the presence of symptoms or changes in blood biochemical and hematological parameters suggestive of a systemic inflammatory state; immunosuppressive treatments ongoing in the last 6 months before inclusion. Motor function assessment included the Children’s Hospital of Philadelphia Infant Test of Neuromuscular Disorders (CHOP INTEND) (Glanzman et al., 2010) for SMA type 1 patients, and the Hammersmith Functional Motor Scale Expanded (HFMSE) (Pera et al., 2017) for SMA type 2 and 3 patients. The study was performed in accordance with the ethical standards of the Declaration of Helsinki. The investigation and use of patients’ data for research purposes were approved by the Fondazione IRCCS Istituto Neurologico Carlo Besta research ethical committee in accordance with the Declaration of the World Medical Association (Project identification code 92/2019, 16 January 2019).

### Serum and cerebrospinal fluid sample collection

Peripheral blood of patients, as well as healthy controls, was drawn on the day of inclusion into the study. All patients and healthy controls were fasting from the previous midnight and did not perform any physical activity before blood and CSF collection, since it has been demonstrated that in response to exercise, some cytokines (e.g., IL-6, TNFα) are released by immune and muscle cells (Ostrowski et al., 1998; Pedersen and Febbraio, 2008; Ball, 2015). Peripheral blood was collected in Greiner Bio-One VACUETTE Z Serum Sep Clot Activator Tubes (Thermo Fisher Scientific, Waltham, MA, United States), centrifuged at 3,000 rpm for 10 min at room temperature. The serum, transferred in cryogenic vials, was immediately stored in liquid nitrogen pending assays. CSF was centrifuged at 3,000 rpm for 10 min at room temperature and collected in cryogenic vials at −80°C. Serum and CSF samples were obtained after patients written consent, or parental written consent in case of pediatric patients, right before first nusinersen infusion (T0, baseline), and after 6 months of treatment (T6). Biological samples were stored at −80°C in the Biobank of Fondazione IRCCS Istituto Neurologico Carlo Besta until use.

### Cytokine quantification

A Bio-Plex Pro-Human Cytokine Immunoassay 96-well kit (Bio-Rad Laboratory, Hercules, CA, United States) was used to measure serum and CSF concentration of the following cytokines on the Bio-Plex 200 (Bio-Rad) system powered by Luminex xMAP technology: interleukin (IL)-1β (IL-1β), IL-4, IL-6, IL-10, IL-17A, IL-17F, IL-21, IL-22, IL-23, IL-31, IL-33, tumor necrosis factor-alpha (TNF-α), and interferon gamma-γ (IFN-γ). Serum samples were diluted 1:4; CSF samples were tested undiluted. Each sample was tested in duplicate, and each plate contained a balanced sample size for the biological groups analyzed. Cytokine concentration was expressed in pg/ml.

### Statistics

Continuous variables are presented as mean and standard deviation, categorical variables as numbers and percentages. Assessment of the normality was performed using Shapiro–Wilk test. Between-groups comparisons of continuous/ordinal variables were performed using two-sample Wilcoxon rank sum test (Mann–Whitney) test. Comparisons among more than two groups were tested using Kruskal–Wallis rank sum test and the *post hoc* Dunn test. We used Spearman’s correlation test to measure association between ordinal and continuous variables. The Pearson’s correlation coefficient was considered for correlations among cytokines. The Pearson’s correlation matrices have been created using R package corrplot. The significant *p*-value threshold was set to 0.05. GraphPad Prism version 4.0 (GraphPad Software, San Diego, CA, United States) and R software were used for graph elaboration.

### Data availability

All anonymized data from this study are stored in the Open Repository of Fondazione IRCCS Istituto Neurologico Carlo Besta, and will be shared on reasonable request from any qualified investigator. Data reuse is permitted only for academic purposes.

## Results

### Demographic and clinical characteristics

Clinical data and bio-samples were collected from 33 SMA patients and adult controls who satisfied the inclusion criteria. Demographic and clinical characteristics of the study population are shown in Table 1. The control group included 8 females and 3 males; mean age at blood collection was 34.9 ± 9.4 years.

**TABLE 1:** Characteristics of the spinal muscular atrophy (SMA) patients participating in the study.

Features	Total (*N* = 33)	Pediatric (*N* = 21)	Adults (*N* = 12)
**Gender**			
F – *n* (%)	19 (57.6%)	12 (57.1%)	7 (53.8%)
**Age at onset (years)**			
Mean ± SD	2.76 ± 4.22	1.02 ± 0.75	5.65 ± 5.89
Median (range)	1.04 (0.25–16)	0.83 (0.25–3)	3 (0.75–16)
**Disease duration at therapy onset (years)**			
Mean ± SD	15.42 ± 18.13	3.39 ± 2.85	35.47 ± 14.52
Median (range)	5.84 (0–53.08)	3 (0–10.83)	37.25 (1–53.08)
**SMA type – *n* (%)**			
1	4 (12.1%)	4 (19%)	0
2	13 (39.4%)	12 (57.1%)	1 (7.7%)
3	16 (48.5%)	5 (23.8%)	11 (84.6%)
***SMN2* copy number – *n* (%)**			
2	10 (30.3%)	10 (47.6%)	0
3	15 (45.5%)	11 (52.4%)	4 (33.3%)
4	8 (24.2%)	0	8 (66.7%)
**SMA maximum motor function at baseline – *n* (%)**			
Non-sitter	5 (15.2%)	4 (19.05%)	1 (8.3%)
Sitter	17 (51.5%)	13 (61.9%)	4 (33.3%)
Walkers	11 (33.3%)	4 (19.05%)	7 (58.3%)
**Motor function scores at baseline**			
**HFMSE**			
Mean ± SD	26.9 ± 19.9	21.3 ± 16.2	34.9 ± 22.5
Median (range)	20 (0–66)	14 (1–57)	44.5 (0–66)
**CHOP INTEND**			
Mean ± SD	31.8 ± 11.2	40.3 ± 14.4	
Median (range)	37 (15–38)	46.5 (19–49)	
**Motor function scores after 6 months of treatment**			
**HFMSE**			
Mean ± SD	29.0 ± 20.02	24.0 ± 17.8	36.1 ± 21.6
Median (range)	21 (0–66)	17 (2–61)	46 (0–66)
**CHOP INTEND**			
Mean ± SD	40.3 ± 14.4	40.3 ± 14.4	
Median (range)	46.5 (19–49)	46.5 (19–49)	

HFMSE, Hammersmith Functional Motor Scale Expanded; CHOP INTEND, Children’s Hospital of Philadelphia Infant Test of Neuromuscular Disorders; SD, standard deviation.

### Increase of cytokine levels in serum of pediatric and adult SMA patients

**FIGURE 1:**
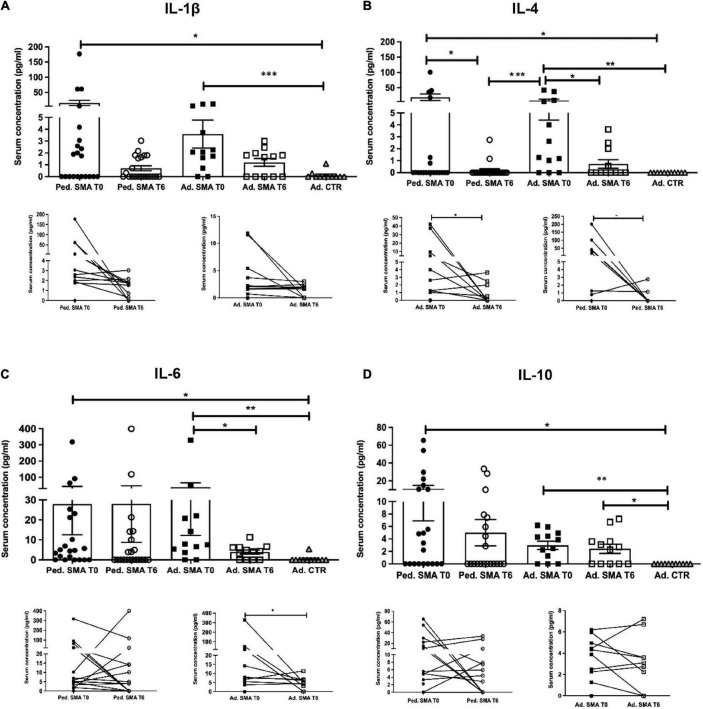
Levels of cytokines in the serum of pediatric and adult spinal muscular atrophy (SMA) patients. The concentration (pg/ml) of **(A)** IL-1β, **(B)** IL-4, **(C)** IL-6, and **(D)** IL-10 was measured in the serum of pediatric (Ped.; circle, *N* = 21) and adult (Ad.; square, *N* = 12) SMA patients at baseline (T0 black) and after 6 months of nusinersen treatment (T6 white), and in healthy controls (triangle) by multiplex immunoassay. Data in the graphs are reported as mean ± SEM for each sample group. The before-after graphs contains the mean cytokine concentration obtained for each patient in the duplicate multiplex immunoassays reactions before and after 6 months of nusinersen treatment (T0 and T6). *p < 0.05, **p < 0.001, *p* < 0.0001*** by Mann–Whitney test.

**FIGURE 2:**
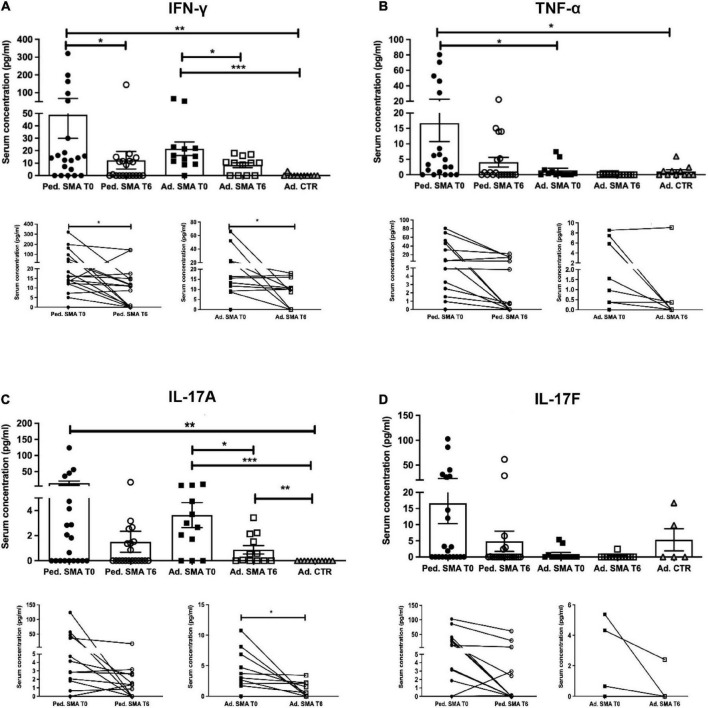
Levels of cytokines in the serum of pediatric and adult spinal muscular atrophy (SMA) patients. The concentration (pg/ml) of **(A)** IFN-γ, **(B)** TNF-α, **(C)** IL-17A, and **(D)** IL-17F was measured in the serum of pediatric (Ped.; circle, *N* = 21) and adult (Ad.; square, *N* = 12) SMA patients at baseline (T0 black) and after 6 months of nusinersen treatment (T6 white), and in healthy controls (gray triangle) by multiplex immunoassay. Data in the graphs are reported as mean ± SEM for each sample group. The before-after graphs contains the mean cytokine concentration obtained for each patient in the duplicate multiplex immunoassays reactions before and after 6 months of nusinersen treatment (T0 and T6). *p < 0.05, **p < 0.001, *p* < 0.0001*** by Mann–Whitney test.

**FIGURE 3:**
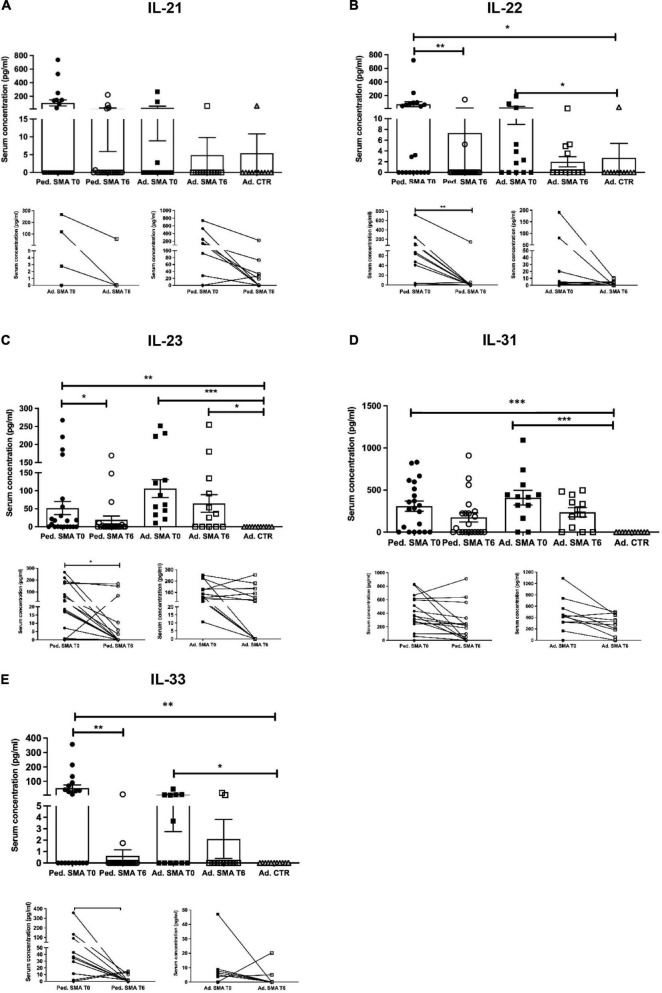
Levels of cytokines in the serum of pediatric and adult spinal muscular atrophy (SMA) patients. The concentration (pg/ml) of **(A)** IL-21, **(B)** IL-22, **(C)** IL-23, **(D)** IL-31, and **(E)** IL-33 was measured in the serum of pediatric (Ped.; circle, *N* = 21) and adult (Ad.; square, *N* = 12) SMA patients at baseline (T0 black) and after 6 months of nusinersen treatment (T6 white), and in healthy controls (gray triangle) by multiplex immunoassay. Data in the graphs are reported as mean ± SEM for each sample group. The before-after graphs contains the mean cytokine concentration obtained for each patient in the duplicate multiplex immunoassays reactions before and after 6 months of nusinersen treatment (T0 and T6). *p < 0.05, **p < 0.001, *p* < 0.0001*** by Mann–Whitney test.

Quantification of serum concentration of 13 pro- and anti-inflammatory cytokines showed a significant increase in the levels of IL-1β (*p* < 0.05), IL-4 (*p* < 0.05), IL-6 (*p* < 0.05), IL-10 (*p* < 0.05), IFN-γ (*p* < 0.01), TNF-α (*p* < 0.05), IL-17A (*p* < 0.01), IL-22 (*p* < 0.05), IL-23 (*p* < 0.001), IL-31 (*p* < 0.001), and IL-33 (*p* < 0.01) in serum of pediatric SMA patients when compared to the adult healthy controls (Figures 1–3). This was supported by data comparison to the reference value ranges reported by Pranzatelli et al. (2013) for pediatric patients affected by non-inflammatory neurological disorders (Supplementary Table 1). In adult SMA patients, we observed a significant increase in serum levels of IL-1β (*p* < 0.001), IL-4 (*p* < 0.01), IL-6 (*p* < 0.01), IL-10 (*p* < 0.01), IFN-γ (*p* < 0.001), IL-17A (*p* < 0.001), IL-22 (*p* < 0.05), IL-23 (*p* < 0.001), IL-31 (*p* < 0.001), and IL-33 (*p* < 0.05), compared to healthy controls (Figures 1–3).

### Decrease of cytokine levels in serum of pediatric and adult SMA patients upon nusinersen treatment

We found a significant decrease in the levels of IL-4 (*p* < 0.05), IFN-γ (*p* < 0.05), IL-22 (*p* < 0.01), IL-23 (*p* < 0.05), and IL-33 (*p* < 0.01), in serum of pediatric SMA patients after 6 months of therapy when compared to baseline values (Figures 1–3). In serum of adult SMA patients, a significant decrease in the levels of IL-4 (*p* < 0.05), IL-6 (*p* < 0.05), INF-γ (*p* < 0.05), and IL-17A (*p* < 0.05) after 6 months of treatment compared to baseline values was found (Figures 1, 2). Notably, levels of significantly reduced cytokines, except for IL-17A, did not differ from adult controls levels, thus they were mostly brought down to normal.

### Detection of inflammatory cytokines in cerebrospinal fluid of pediatric and adult SMA patients

**FIGURE 4:**
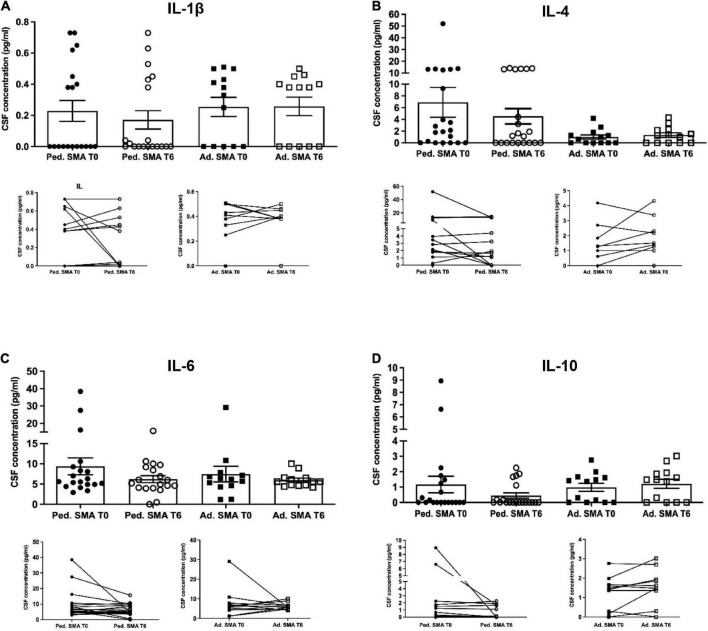
Levels of cytokines in cerebrospinal fluid (CSF) of pediatric and adult spinal muscular atrophy (SMA) patients. The concentration (pg/ml) of **(A)** IL-1β, **(B)** IL-4, **(C)** IL-6, and **(D)** IL-10 was measured in the serum of pediatric (Ped.; circle, *N* = 21) and adult (Ad.; square, *N* = 13) SMA patients at baseline (T0 black) and after 6 months of nusinersen treatment (T6 white), and in healthy control (Ad. CTR; gray triangle) by multiplex immunoassay. Data in the graphs are reported as mean ± SEM for each sample group. The before-after graphs contains the mean cytokine concentration obtained for each patient in the duplicate multiplex immunoassays reactions before and after 6 months of nusinersen treatment (T0 and T6). *p* > 0.05, Mann–Whitney test.

**FIGURE 5:**
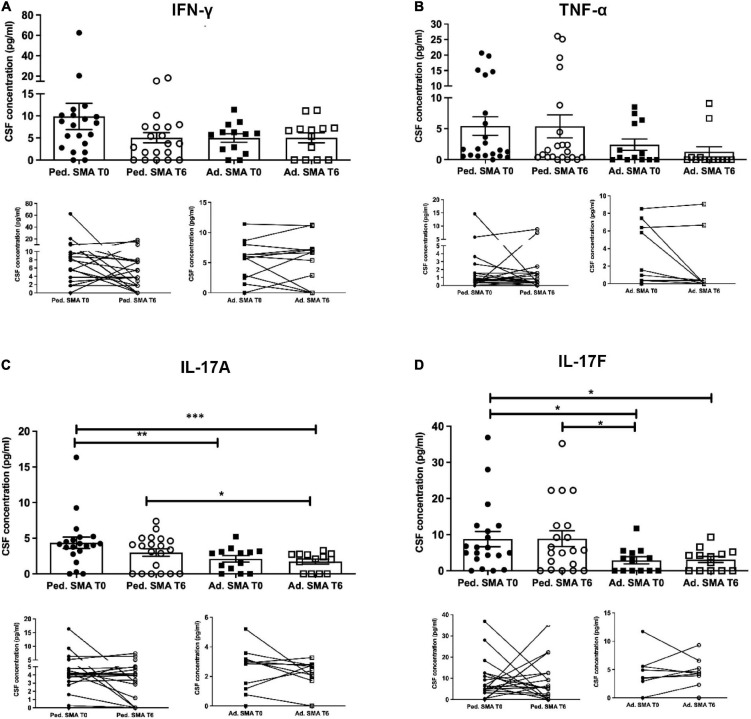
Levels of cytokines in cerebrospinal fluid (CSF) of pediatric and adult spinal muscular atrophy (SMA) patients. The concentration (pg/ml) of levels of **(A)** IFN-γ, **(B)** TNF-α, **(C)** IL-17A, and **(D)** IL-17F were measured in the serum of pediatric (Ped.; circle, *N* = 21) and adult (Ad.; square, *N* = 13) SMA patients at baseline (T0 black) and after 6 months of nusinersen treatment (T6 white), and in healthy controls (Ad. CTR; gray triangle) by multiplex immuno assay. Data in the graphs are reported as mean ± SEM for each sample group. The before-after graphs contains the mean cytokine concentration obtained for each patient in the duplicate multiplex immunoassays reactions before and after 6 months of nusinersen treatment (T0 and T6). *p < 0.05, **p < 0.001, *p* < 0.0001*** by Mann–Whitney test.

**FIGURE 6:**
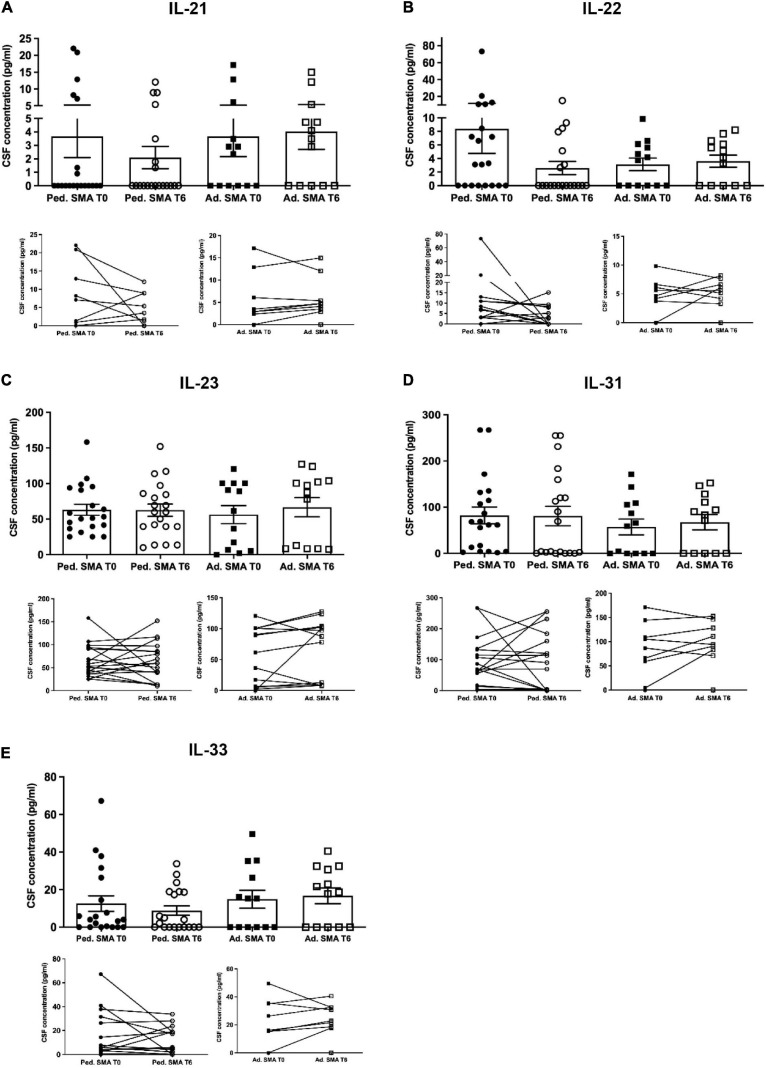
Levels of cytokines in cerebrospinal fluid (CSF) of pediatric and adult spinal muscular atrophy (SMA) patients. The concentration (pg/ml) of levels of **(A)** IL-21, **(B)** IL-22, **(C)** IL-23, **(D)** IL-31, and **(E)** IL-33 were measured in the serum of pediatric (Ped.; circle, *N* = 21) and adult (Ad.; square, *N* = 13) SMA patients at baseline (T0 black) and after 6 months of nusinersen treatment (T6 white), and in healthy controls (Ad. CTR; gray triangle) by multiplex immuno assay. Data in the graphs are reported as mean ± SEM for each sample group. The before-after graphs contains the mean cytokine concentration obtained for each patient in the duplicate multiplex immunoassays reactions before and after 6 months of nusinersen treatment (T0 and T6). *p* > 0.05, Mann–Whitney test.

We observed that CSF of both pediatric and adult SMA patients contained detectable levels of the 13 cytokines (Figures 4–6). Interestingly, IL-17A levels were significantly increased in pediatric patients compared to adult patients at baseline (*p* < 0.01) and after treatment (*p* < 0,05). IL-17F levels were significantly increased in pediatric patients compared to adult patients at baseline (*p* < 0.05), and after treatment levels were still increased compared to the ones of adults before treatment (*p* < 0.05) (Figure 5). Cytokine increase in pediatric patients was confirmed by data comparison with the reference value ranges reported by Pranzatelli et al. (2013) for children affected by non-inflammatory neurological disorders (Supplementary Table 1). CSF cytokine levels did not show significant changes after 6 months of nusinersen therapy compared to the baseline levels in both pediatric and adult SMA patients (Figures 4–6).

### Identification of correlations among serum and cerebrospinal fluid cytokines, clinical score of motor function, and *SMN2* copy number

**FIGURE 7:**
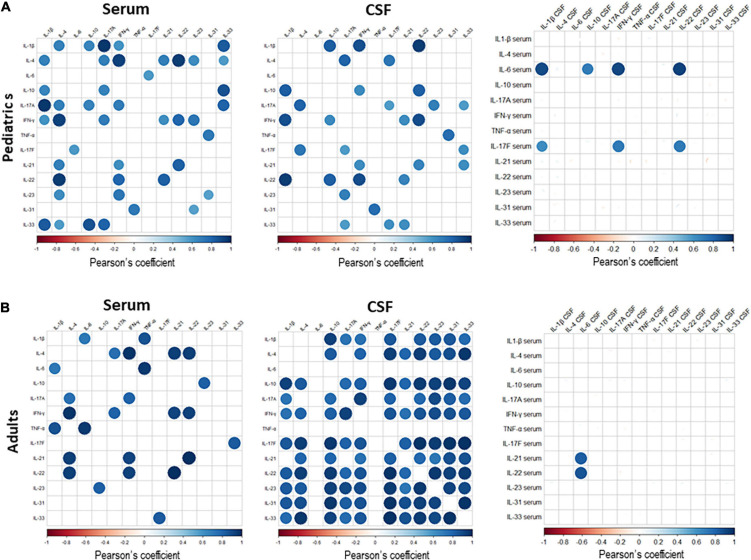
The correlation matrix among all cytokines expressed in serum and cerebrospinal fluid (CSF) of pediatric **(A)** and adult **(B)** Spinal muscular atrophy (SMA) patients. Pearson’s correlation coefficients r ≥0.5 or ≤0.5 are shown. White squares lack statistical significance (*p* > 0.05). The color in each circle indicates the Pearson’s correlation coefficient r among the variables reported in the two coordinates, as indicated by colored scale bar.

In order to investigate the relationship among the different cytokines tested in serum and CSF, and specifically to assess whether their levels were correlated with each other, we performed Pearsons’ correlation analyses in pediatric and adult SMA patients at baseline. We observed a positive correlation between different cytokines in serum (Figure 7), with the stronger correlation found for the following cytokine pairs: (1) IL-17a and IL-1β, IFN-γ and IL-4, IL-22 and IL-4, in pediatric patients; and (2) IFN-γ and IL-4, TNF-α and IL-6, IL-21 and IL-22, in adult patients.

In CSF, cytokine pairs showing a strong positive correlation were: (1) IL-1β and IL-22, IL-1 β and IFN- γ, IFN- γ and IL-22, in pediatric patients; and (2) IL-1β and IL-10; IL-4 with IL-17F, IL-22, and IL-33; IL-10 with IL-17F, IL-22, IL-23, IL-31 and IL-33; IL 17a and IFN-γ; IL-17F with -IL-22, IL-23, IL-31 and IL-33; IL-22 with IL-23, IL-31 and IL-33, in adult patients (Figure 7).

We next performed correlation analyses between the cytokine levels in serum and those in CSF. In pediatric patients, we observed that serum IL-6 levels positively correlated with those of IL1-β, IL-10, IFN-γ and IL-22 in CSF, and serum IL-17F levels positively correlated with those of IL-1 β, IFN-γ and IL-22 in CSF. In adults, a positive correlation was observed between serum levels of IL-21 and IL-22 with levels of IL-6 in CSF (Figure 7). Correlation among the levels of the different cytokines tested is indicative of a common mechanism related to their production and associated with SMA.

To test whether the identified cytokine levels could be related to different clinical response to therapy, we performed regression analyses among serum and CSF cytokine levels with HFMSE score at baseline and after treatment, and between cytokine levels with ΔHFMSE (i.e., HFMSE score post treatment – HFMSE pretreatment). No correlation analysis with CHOP INTEND was performed due to the small number of SMA type 1 patients. We found that lower serum levels of IL-23 at baseline were positive predictors of a better clinical outcome (ΔHFMSE) after 6 months of nusinersen therapy in pediatric patients (Spearman r_s_ = −0.53, *p* = 0.029). We also found that, after 6 months of nusinersen therapy, IL-10 expression in pediatric serum positively correlated with a better HFMSE score (Sperman r_s_ = 0.67, *p* = 0.003).

No correlation among cytokines levels and HFMSE score at baseline and after 6 months, or ΔHFMSE, was detected if the analysis was corrected for SMA type, months at onset and baseline motor milestone, both in pediatric and adult patient.

Correlation analyses among cytokine levels at baseline and *SMN2* copy numbers demonstrated a significant difference between patients carrying two and four copies for: IL-4 (*p* = 0.021) and IL-23 (*p* = 0.009) in serum, where a higher concentration of cytokine corresponded to a higher *SMN2* copy number; TNF-α (*p* = 0.036), IL-31 (*p* = 0.013), and IFN-γ (*p* = 0.015) in CSF of patients, where a higher cytokine level correlated with a lower number of copies. A significant correlation among patients carrying three and four copies of *SMN2* and cytokine expression at baseline was detected for: IL-4 (*p* = 0.005), IL-17A (*p* = 0.015), and IL-17-F (*p* = 0.010) in CSF, with higher cytokine levels in patients carrying lower *SMN2* copy numbers. After 6 months of nusinersen treatment, IL-31 expression in CSF of SMA patients with two copies of *SMN2* was higher compared to patients carrying three copies (*p* = 0.011) and four copies (*p* = 0.016). Similarly, higher CSF levels of TNF-α and IL-17F distinguished SMA patients with two copies of *SMN2* from patients with four copies (*p* = 0.009 and *p* = 0.035, respectively), and patients with three copies of *SMN2* from patients with four copies (*p* = 0.047 and *p* = 0.060, respectively).

## Discussion

Multisystem involvement in SMA has gained prominence since different therapeutic options are emerging, making the way for new SMA phenotypes and consequent challenges in clinical care. Defective immune organs have been found in preclinical models of SMA (Deguise and Kothary, 2017), suggesting an involvement of the immune system in the disease. However, the immune state in SMA patients has not been investigated so far. Here, we aimed to longitudinally assess the levels of innate and adaptive immune factors, selected for their associated with innate immunity and adaptive immune cell populations including T-helper (Th) 1, Th2, and Th17 cell subtypes, in serum and CSF of pediatric and adult SMA patients pre- and post-nusinersen therapy.

Overall, we found a cytokine inflammatory signature in both serum and CSF of pediatric and adult SMA patients, suggestive of inflammation and immune system activation at a peripheral and central level in our patients’ cohort.

One striking finding was that serum of both pediatric and adult SMA patients displayed increased levels of IFN-γ, IL-17a and IL-22, which are signature cytokines of activated Th1/Th17cells (Damsker et al., 2010). Along with a crucial role in host defense, these cells are known to be key players in the pathogenesis of different autoimmune diseases (Tesmer et al., 2008). Indeed, they sustain pro-inflammatory T cell-mediated responses by producing inflammatory cytokines, that in turn activate innate and adaptive immune system cells. Of note, sustained inflammation caused by Th1/Th17 pathway activation has been, recently, related to disease activity in neurodegenerative diseases (Zhang et al., 2013; Storelli et al., 2019), particularly in amyotrophic lateral sclerosis (Jin et al., 2020).

Spinal muscular atrophy is a heterogeneous disease, ranging from *in utero* to adult-onset forms (Wirth, 2021). Our study has been developed in a real-world environment, starting from the advent of nusinersen, thus it includes a wide age range, reflecting SMA epidemiology. In agreement with an expected more active disease in pediatric patients compared to adult, mostly milder and chronic, SMA forms (Yeo and Darras, 2020), we found that at baseline (i.e., before nusinersen treatment) the levels of TNF-α, a pivotal cytokine released from Th1 and Th17 cells, was increased only in pediatric patients’ sera. In the same subset, we showed a positive correlation between IL-1ß and IL-17a, and between TNF-α and IL-31, another cytokine strictly related to Th17 cells (Bautista-Herrera et al., 2020).

In line with the Th17 signature observed in SMA serum, we also detected increased serum levels of IL-1ß, IL-6, and IL-23 in pediatric and adult patients, which are important promoter of Th1/Th17 development and maintenance (Langrish et al., 2005; Acosta-Rodriguez et al., 2007; Villegas et al., 2019). Indeed, IL-1ß and IL-6 are prominent inflammatory response mediator released by macrophages (Dinarello, 2018); IL-23 is released by dendritic cells (DCs) and macrophages and, together with other Th17-related cytokines, constitutes a pathway able to chronically sustain inflammation in several inflammatory autoimmune diseases, to the extent that it has been considered as a new therapeutic target for these disorders (Tan et al., 2009). Interestingly, IL-33, a cytokine released from DCs known to induce Th17 cells *via* IL-1β and IL-6, was increased in pediatrics’ and adults’ sera, where it was positively correlated with IL-17A and IL-17F cytokines, respectively (Park et al., 2017).

We also revealed an increase in IL-10 concentration in sera of our population. IL-10 is an anti-inflammatory cytokine produced by different cell types, such as T-regulatory cells (Tregs) and Th2 cells. A positive feedback loop has been described for Th1 cells, which would produce IL-10 themselves to limit their pro-inflammatory activity (Trinchieri, 2007), further supporting a Th1 signature in SMA.

Taken together, all these data suggest the engagement of the Th1/Th17 pathway in SMA, both in pediatric and adult population.

Interestingly, after 6 months of nusinersen administration, most of the cytokines implicated in the Th1/Th17 signature were significantly reduced in patients’ serum. This response to therapy was slightly more evident in pediatric patients, where IFN- γ, IL-22, IL-23, and IL-33 levels were significantly lower compared to baseline, while in adults IL-6, and IL-17a decreased after therapy together with IFN- γ. Also, IL-4 and IL-23 serum levels that distinguished, before treatment, patients with four *SMN2* copy numbers from patients carrying two copies, no longer showed any difference after 6 months of nusinersen, suggesting a “normalizing effect” of the therapy across disease subgroups. Since IL-4 is known for its anti-inflammatory properties and may be produced by activated immune system cells as an attempt to counteract inflammation, its reduction after the therapy may reflect generalized reduction in immune activation as an effect of nusinersen.

By linear regression analysis we found that a higher baseline expression of IL-23 in serum correlated with a worse motor function outcome after treatment in pediatric patients. This significance further improves considering only SMA type 2 population, a more severe disease form compared to SMA type 3. This points out a possible role for IL-23 as predictive biomarkerof response to therapy. We also found that, after 6 months of treatment, pediatric patients presenting a higher IL-10 concentration on serum showed a better HFMSE score, which is in line with IL-10 protective role, and makes IL-10 worthy of attention as a potential pharmacodynamics biomarker.

Although nusinersen is directly administered within the central nervous system (CNS) by intrathecal injection, and it is distributed mostly in the target spinal cord tissue, our findings suggest a potential beneficial effect of the drug on peripheral immune system that might be monitored by cytokine assessment in the serum. This hypothesis is supported by evidence of nusinersen in peripheral tissues, as liver, kidney, and muscle (Europa EU, 2017), which suggests that its effect on peripheral inflammation/immune dysregulation may be due to a direct impact on immune cells/organs rather than the indirect result of its intrathecal distribution.

In agreement with the emerging evidence that various systemic aspects may be altered in SMA, and may benefit from nusinersen therapy, we also revealed a significant reduction of IL-4 in serum after treatment. Of note, IL-4 is involved in direct activation of sensory neurons in atopic inflammation and, together with IL-31, represents a link between innate and adaptive immune system and, in turn, between immune system and the peripheral nervous system (Kader et al., 2021). Recently, it has been hypothesized that these cytokines display regulatory effects on neuronal physiology of the sensory nervous system (Oetjen and Kim, 2018), which is of relevance since it is emerging that the sensory system, and particularly the proprioceptive synapses, is defective in SMA and contributes to motor neurons disfunction (Shorrock et al., 2019).

Concurrently to serum evidence, we were able to detect the majority of the cytokines at pathological levels also in the CSF of our SMA patients. Unfortunately, CSF samples, or datasets of cytokine concentrations in CSF, from control subjects, such as patients with non-inflammatory neurological disorders, were not available. Comparison of our pediatric CSF data with cytokine reference range reported by Pranzatelli et al. (2013) for children without inflammatory CNS diseases suggests abnormally increased cytokine levels in our pediatric SMA patients. Cytokines’ concentrations were overall lower compared to those observed in serum, except for the noticeable levels of IL-31 and IL-23. However, the observation of a pro-inflammatory cytokine (e.g., IL-17A, IL-23, IL-31, IL-33, TNF-α) profile in CSF of SMA patients is relevant and indicative of a pathological inflammatory state. Strong correlations were found between levels of IFN-γ and those of IL-1β and IL-22, and between IL-17A levels and those of IL-23, in both pediatric and adult CSFs, which suggests an activation of the Th1/Th17 cytokine profile also in the CNS. Interestingly, as learned from other CNS disorders, Th1/Th17 cells target microglia and astrocytes, promoting neuroinflammation (Prajeeth et al., 2017). This is in line with previous studies demonstrating a glial activation in SMA patients’ spinal cords (Brock and McIlwain, 1984; Kuru et al., 2009) where astrocyte-specific SMN restoration resulted in IL-1 β and IL-6 cytokine decrease (Rindt et al., 2015). Correlation analyses of CSF cytokine levels with *SMN2* copy numbers demonstrated that patients carrying two or three *SMN2* copies (nearly all pediatrics) presented higher levels of the differently expressed cytokines compared to patients with four *SMN2* copies (entirely adults), underlining a more active immunological environment in CNS of pediatric patients. Surprisingly, considered the direct delivery of nusinersen in the CSF, 6 months after treatment we did not observe a significant reduction, consistent across patients, in any of the cytokines investigated. In this regard, we may speculate that modifications of cytokine pattern in the serum but not in the CSF after 6 months of nusinersen treatment, may reflect a more precocious effect on peripheral immunological aspects, compared to the CNS.

Whether *SMN1* deficiency impacts immune organs, and if immune system activation or motor neuron injury leads to the release of specific antigens which induce priming of Th1/Th17 cells need to be clarified. Relying on our data, we did not find correlations between serum and CSF levels for each cytokine, at baseline and after treatment, neither relevant associations with laboratory parameters related to blood-brain barrier permeability (data not shown). All these data are not in favor of a cytokine leakage from periphery to CNS. More extensive investigations are required to further evaluate neuroinflammation in SMA patients.

The longitudinal assessment of immune soluble factors proposed, pre- and post-nusinersen therapy, has never been ruled out in SMA patients. Here, we demonstrated an inflammatory peripheral signature, that changes upon the *SMN2* modulating treatment, and the presence of inflammatory mediators in CSF of SMA patients, thus supporting an inflammatory/immunological contribution to SMA. Of note, we provide evidence of a possible role for serum IL-23 as predictive biomarkers of response to nusinersen therapy, and of serum IL-10 as a potential on-treatment monitoring biomarker.

Despite a relatively small cohort which does not allow stratification analyses, and the short-term follow-up, we were able to detect statistical significant differences in the peripheral pro-inflammatory profile between pediatric and adult patients, and after treatment. The outcomes of our investigation pave the way toward further studies in larger patients’ cohorts, promising to get relevant insights into the immunological contribution to SMA, for their translation into the clinical practice. The inflammatory molecules here identified could indeed represent novel potential therapeutic targets, as well as reliable biomarkers useful to stratify patients, predict disease progression and monitor response to therapies, for a better management of SMA patients.

## Data availability statement

The original contributions presented in the study are included in the article/Supplementary material, further inquiries can be directed to the corresponding authors.

## Ethics statement

The studies involving human participants were reviewed and approved by Fondazione IRCCS Istituto Neurologico Carlo Besta Research Ethical Committee. Written informed consent to participate in this study was provided by the participants’ legal guardian/next of kin.

## Author contributions

SB performed and supervised the clinical activities during nusinersen treatments in adults, provided sample acquisition, performed data analysis and their interpretation, and drafted the manuscript and the figures. PC contributed to conception and design of the study, acquisition, and analysis of data. ES performed statistical analysis of data and contributed to their interpretation. EG, CM, and MC performed data acquisition. FA collected clinical samples. AV, VP, and CG performed genetic analysis and critically revised the manuscript. RZ and MA collected clinical data and performed HFMSE and CHOP evaluations. CD and RiM performed and supervised the clinical activities during nusinersen treatments in children, provided sample acquisition and critically revised the manuscript. ReM supervised the clinical and research activity. LM supervised the clinical activities during nusinersen treatments in adults, contributed to interpretation of data, and revised the manuscript. SM performed the conception and design of the study, the acquisition and analysis of data, and the drafting of the manuscript and figures. All authors read, revised, and approved the final manuscript.

## References

[B1] Acosta-Rodriguez E. V. Napolitani G. Lanzavecchia A. Sallusto F. (2007). Interleukins 1beta and 6 but not transforming growth factor-beta are essential for the differentiation of interleukin 17-producing human T helper cells. *Nat. Immunol.* 8 942–949. 10.1038/ni1496 17676045

[B2] Ball D. (2015). Metabolic and endocrine response to exercise: Sympathoadrenal integration with skeletal muscle. *J. Endocrinol.* 224 R79–R95. 10.1530/JOE-14-0408 25431226

[B3] Bautista-Herrera L. A. De la Cruz-Mosso U. Román-Fernández I. V. Parra-Rojas I. Soñanez-Organis J. G. Hernández-Bello J. (2020). A potential inflammatory role of IL-31 in psoriatic arthritis: A correlation with Th17 cytokine profile. *Int. J. Immunopathol. Pharmacol.* 34:2058738420907186. 10.1177/2058738420907186 32138573 PMC7065432

[B4] Brock T. O. McIlwain D. L. (1984). Astrocytic proteins in the dorsal and ventral roots in amyotrophic lateral sclerosis and Werdnig-Hoffmann disease. *J. Neuropathol. Exp. Neurol.* 43 609–619. 10.1097/00005072-198411000-00005 6502190

[B5] Chaytow H. Faller K. M. E. Huang Y.-T. Gillingwater T. H. (2021). Spinal muscular atrophy: From approved therapies to future therapeutic targets for personalized medicine. *Cell Rep. Med.* 2:100346. 10.1016/j.xcrm.2021.100346 34337562 PMC8324491

[B6] Chaytow H. Huang Y. T. Gillingwater T. H. Faller K. M. E. (2018). The role of survival motor neuron protein (SMN) in protein homeostasis. *Cell. Mol. Life Sci.* 75 3877–3894. 10.1007/s00018-018-2849-1 29872871 PMC6182345

[B7] Chiriboga C. A. (2017). Nusinersen for the treatment of spinal muscular atrophy. *Expert Rev. Neurother.* 17 955–962. 10.1080/14737175.2017.1364159 28884620

[B8] Crisafulli S. G. Brajkovic S. Cipolat Mis M. S. Parente V. Corti S. (2018). Therapeutic strategies under development targeting inflammatory mechanisms in amyotrophic lateral sclerosis. *Mol. Neurobiol.* 55 2789–2813. 10.1007/s12035-017-0532-4 28455693

[B9] Damsker J. M. Hansen A. M. Caspi R. R. (2010). Th1 and Th17 cells: Adversaries and collaborators. *Ann. N. Y. Acad. Sci.* 1183 211–221. 10.1111/j.1749-6632.2009.05133.x 20146717 PMC2914500

[B10] Darras B. T. Masson R. Mazurkiewicz-Bełdzińska M. Rose K. Xiong H. Zanoteli E. (2021). Risdiplam-treated infants with type 1 spinal muscular atrophy versus historical controls. *N. Engl. J. Med.* 385 427–435. 10.1056/NEJMoa2102047 34320287

[B11] Deguise M. O. Kothary R. (2017). New insights into SMA pathogenesis: immune dysfunction and neuroinflammation. *Ann. Clin. Transl. Neurol.* 4 522–530. 10.1002/acn3.423 28695153 PMC5497530

[B12] Dinarello C. A. (2018). Overview of the IL-1 family in innate inflammation and acquired immunity. *Immunol. Rev.* 281 8–27. 10.1111/imr.12621 29247995 PMC5756628

[B13] Europa EU (2017). *Spinraza, INN-nusinersen.* Available online at: https://ec.europa.eu/health/documents/communityregister/2017/20170530137918/anx_137918_en.pdf (accessed June 30, 2022).

[B14] Finkel R. S. Mercuri E. Darras B. T. Connolly A. M. Kuntz N. L. Kirschner J. (2017). Nusinersen versus Sham control in infantile-onset spinal muscular atrophy. *N. Engl. J. Med.* 377 1723–1732. 10.1056/NEJMoa1702752 29091570

[B15] Finkel R. Bertini E. Muntoni F. Mercuri E. (2015). ENMC SMA workshop study group. 209th ENMC international workshop: Outcome measures and clinical trial readiness in spinal muscular atrophy 7–9 November 2014, Heemskerk, The Netherlands. *Neuromuscul. Disord.* 25 593–602. 10.1016/j.nmd.2015.04.009 26045156

[B16] Glanzman A. M. Mazzone E. Main M. Pelliccioni M. Wood J. Swoboda K. J. (2010). The children’s hospital of Philadelphia infant test of neuromuscular disorders (CHOP INTEND): Test development and reliability. *Neuromuscul. Disord.* 20 155–161. 10.1016/j.nmd.2009.11.014 20074952 PMC3260046

[B17] Hamilton G. Gillingwater T. H. (2013). Spinal muscular atrophy: Going beyond the motor neuron. *Trends Mol. Med*. 19 40–50. 10.1016/j.molmed.2012.11.002 23228902

[B18] Jin M. Günther R. Akgün K. Hermann A. Ziemssen T. (2020). Peripheral proinflammatory Th1/Th17 immune cell shift is linked to disease severity in amyotrophic lateral sclerosis. *Sci. Rep.* 10:5941. 10.1038/s41598-020-62756-8 32246039 PMC7125229

[B19] Kader H. A. Azeem M. Jwayed S. A. Al-Shehhi A. Tabassum A. Ayoub M. A. (2021). Current insights into immunology and novel therapeutics of atopic dermatitis. *Cells* 10:1392. 10.3390/cells10061392 34200009 PMC8226506

[B20] Kuru S. Sakai M. Konagaya M. Yoshida M. Hashizume Y. Saito K. (2009). An autopsy case of spinal muscular atrophy type III (Kugelberg-Welander disease). *Neuropathology* 29 63–67. 10.1111/j.1440-1789.2008.00910.x 18410269

[B21] Langrish C. L. Chen Y. Blumenschein W. M. Mattson J. Basham B. Sedgwick J. D. (2005). IL-23 drives a pathogenic T cell population that induces autoimmune inflammation. *J. Exp. Med.* 201 233–240. 10.1084/jem.20041257 15657292 PMC2212798

[B22] Maggi L. Bello L. Bonanno S. Govoni A. Caponnetto C. Passamano L. (2020). Nusinersen safety and effects on motor function in adult spinal muscular atrophy type 2 and 3. *J. Neurol. Neurosurg. Psychiatry* 91 1166–1174. 10.1136/jnnp-2020-323822 32917822

[B23] Mendell J. R. Al-Zaidy S. Shell R. Arnold W. D. Rodino-Klapac L. R. Prior T. W. (2017). Single-dose gene-replacement therapy for spinal muscular atrophy. *N. Engl. J. Med.* 377 1713–1722. 10.1056/NEJMoa1706198 29091557

[B24] Mercuri E. Bertini E. Iannaccone S. T. (2012). Childhood spinal muscular atrophy: Controversies and challenges. *Lancet Neurol.* 11 443–452. 10.1016/S1474-4422(12)70061-3 22516079

[B25] Mercuri E. Muntoni F. Baranello G. Masson R. Boespflug-Tanguy O. Bruno C. (2021). Onasemnogene abeparvovec gene therapy for symptomatic infantile-onset spinal muscular atrophy type 1 (STR1VE-EU): An open-label, single-arm, multicentre, phase 3 trial. *Lancet Neurol.* 20 832–841. 10.1016/S1474-4422(21)00251-9 34536405

[B26] Migaj M. Janowicz W. Krajewska G. Bernatowska E. Madaliński K. (1986). Evaluation of cell-mediated and humoral immunity in children suffering from spinal muscular atrophy. *Arch. Immunol. Ther. Exp. (Warsz)* 34 561–567. 3496065

[B27] Oetjen L. K. Kim B. S. (2018). Interactions of the immune and sensory nervous systems in atopy. *FEBS J.* 285 3138–3151. 10.1111/febs.14465 29637705 PMC6516504

[B28] Ostrowski K. Rohde T. Zacho M. Asp S. Pedersen B. K. (1998). Evidence that interleukin-6 is produced in human skeletal muscle during prolonged running. *J. Physiol.* 508 949–953. 10.1111/j.1469-7793.1998.949bp.x 9518745 PMC2230908

[B29] Park S. H. Kim M. S. Lim H. X. Cho D. Kim T. S. (2017). IL-33-matured dendritic cells promote Th17 cell responses *via* IL-1β and IL-6. *Cytokine* 99 106–113. 10.1016/j.cyto.2017.07.022 28802996

[B30] Pedersen B. K. Febbraio M. A. (2008). Muscle as an endocrine organ: Focus on muscle-derived interleukin-6. *Physiol. Rev.* 88 1379–1406. 10.1152/physrev.90100.2007 18923185

[B31] Pera M. C. Coratti G. Forcina N. Mazzone E. S. Scoto M. Montes J. (2017). Content validity and clinical meaningfulness of the HFMSE in spinal muscular atrophy. *BMC Neurol.* 17:39. 10.1186/s12883-017-0790-9 28231823 PMC5324197

[B32] Prajeeth C. K. Kronisch J. Khorooshi R. Knier B. Toft-Hansen H. Gudi V. (2017). Effectors of Th1 and Th17 cells act on astrocytes and augment their neuroinflammatory properties. *J. Neuroinflammation* 14:204. 10.1186/s12974-017-0978-3 29037246 PMC5644084

[B33] Pranzatelli M. R. Tate E. D. McGee N. R. Colliver J. A. (2013). Pediatric reference ranges for proinflammatory and anti-inflammatory cytokines in cerebrospinal fluid and serum by multiplexed immunoassay. *J. Interferon Cytokine Res.* 33 523–528. 10.1089/jir.2012.0132 23659672 PMC3760063

[B34] Rindt H. Feng Z. Mazzasette C. Glascock J. J. Valdivia D. Pyles N. (2015). Astrocytes influence the severity of spinal muscular atrophy. *Hum. Mol. Genet.* 24 4094–4102. 10.1093/hmg/ddv148 25911676 PMC5007659

[B35] Ryniewicz B. Pawińska M. (1978). Preliminary immunological studies in spinal muscular atrophy. *Eur. J. Pediatr.* 128 57–60. 10.1007/BF00496927 668717

[B36] Shorrock H. K. Gillingwater T. H. Groen E. J. N. (2019). Molecular mechanisms underlying sensory-motor circuit dysfunction in SMA. *Front. Mol. Neurosci.* 12:59. 10.3389/fnmol.2019.00059 30886572 PMC6409332

[B37] Storelli E. Cassina N. Rasini E. Marino F. Cosentino M. (2019). Do Th17 lymphocytes and IL-17 contribute to Parkinson’s disease? A systematic review of available evidence. *Front. Neurol.* 10:13. 10.3389/fneur.2019.00013 30733703 PMC6353825

[B38] Tan Z. Y. Bealgey K. W. Fang Y. Gong Y. M. Bao S. (2009). Interleukin-23: Immunological roles and clinical implications. *Int. J. Biochem. Cell Biol.* 41 733–735. 10.1016/j.biocel.2008.04.027 18725317

[B39] Tesmer L. A. Lundy S. K. Sarkar S. Fox D. A. (2008). Th17 cells in human disease. *Immunol. Rev.* 223 87–113. 10.1111/j.1600-065X.2008.00628.x 18613831 PMC3299089

[B40] Tosolini A. P. Sleigh J. N. (2017). Motor neuron gene therapy: Lessons from spinal muscular atrophy for amyotrophic lateral sclerosis. *Front. Mol. Neurosci.* 10:405. 10.3389/fnmol.2017.00405 29270111 PMC5725447

[B41] Trinchieri G. (2007). Interleukin-10 production by effector T cells: Th1 cells show self control. *J. Exp. Med.* 204 239–243. 10.1084/jem.20070104 17296790 PMC2118719

[B42] Villegas J. A. Bayer A. C. Ider K. Bismuth J. Truffault F. Roussin R. (2019). Il-23/Th17 cell pathway: A promising target to alleviate thymic inflammation maintenance in myasthenia gravis. *J. Autoimmun.* 98 59–73. 10.1016/j.jaut.2018.11.005 30578016

[B43] Wan B. Feng P. Guan Z. Sheng L. Liu Z. Hua Y. (2018). A severe mouse model of spinal muscular atrophy develops early systemic inflammation. *Hum. Mol. Genet.* 27 4061–4076. 10.1093/hmg/ddy300 30137324

[B44] Wirth B. (2021). Spinal muscular atrophy: In the challenge lies a solution. *Trends Neurosci.* 44 306–322. 10.1016/j.tins.2020.11.009 33423791

[B45] Yeo C. J. J. Darras B. T. (2020). Overturning the paradigm of spinal muscular atrophy as just a motor neuron disease. *Pediatr. Neurol.* 109 12–19. 10.1016/j.pediatrneurol.2020.01.003 32409122

[B46] Zhang J. Ke K. F. Liu Z. Qiu Y. H. Peng Y. P. (2013). Th17 cell-mediated neuroinflammation is involved in neurodegeneration of aβ1-42-induced Alzheimer’s disease model rats. *PLoS One* 8:e75786. 10.1371/journal.pone.0075786 24124514 PMC3790825

