# Supplementary material for: Identification of a cytokine profile in serum and cerebrospinal fluid of pediatric and adult spinal muscular atrophy patients and its modulation upon nusinersen treatment
Source: Front Cell Neurosci. 2022 Aug 11;16:982760. doi: 10.3389/fncel.2022.982760 (PMC9406526; doi:10.3389/fncel.2022.982760)
Supplement: Supplementary file 1 [file Table_1.docx]

Table S1. Cytokine concentrations in pediatric SMA patients compared to reference values.

| **Cytokine**^(a)^ | **Concentration in our pediatric SMA patients**  (pg/ml) | | **Concentration in pediatric patients with non-inflammatory neurological disorders reported by Pranzatelli et al.^25^**  (pg/ml) | |
| --- | --- | --- | --- | --- |
|  | **Serum**  Means (SD) | **CSF**  Means (SD) | **Serum**  Means (SD) | **CSF**  Means (SD) |
| IL-1β | 15.34 (41.13) | 0.35 (0.63) | undetectable | undetectable |
| IL-4 | 19.04 (48.17) | 7.80 (11.69) | <15 (31) | <5 (3) |
| IL-6 | 29.18 (71.98) | 100.44 (407.36) | ~5 (10) | ~10 (11) |
| IL-10 | 10.89 (18.22) | 1.17 (2.39) | undetectable | ~5 (29) |
| IFN-γ | 47.13 (82.72) | 22.73 (5.40) | ~5 (6) | <5 (3) |
| TNF-α | 11.85 (20.24) | 5.67 (6.97) | undetectable | undetectable |
| IL-17A | 13,37 (30,18) | 4.37 (3.56) | N.A. | N.A. |
| IL-17F | 16.62 (28.82) | 8.78 (9.49) | N.A. | N.A. |
| IL-21 | 98.31 (193.89) | 2.76 (5.90) | N.A. | N.A. |
| IL-22 | 69.91 (160.09) | 8.39 (16.30) | N.A. | N.A. |
| IL-23 | 52.01 (83.36) | 63.25 (36.61) | N.A. | N.A. |
| IL-31 | 308.43 (281.61) | 82.25 (81.26) | N.A. | N.A. |
| IL-33 | 48.64 (89.34) | 20.26 (20.96) | N.A. | N.A. |

^(a)^ Reference values (Pranzatelli et al., 2013) were available for 6 of the 13 cytokines tested in our study.
